# Detoxification of Aflatoxin B1 Contaminated Maize Using Human CYP3A4

**DOI:** 10.4014/jmb.2003.03032

**Published:** 2020-05-13

**Authors:** Marie Yamada, Koji Hatsuta, Mayuko Niikawa, Hiromasa Imaishi

**Affiliations:** Division of Signal Responses, Biosignal Research Center, Kobe University, Nada, Kobe 657-8501, Japan

**Keywords:** Aflatoxin B1, cytochrome P450, CYP3A4, detoxification

## Abstract

Aflatoxin B1 (AFB1) is a mycotoxin produced by *Aspergillus flavus* (*A. flavus*). AFB1 is reported to have high thermal stability and is not decomposed by heat treatment during food processing. Therefore, in this study, knowing that AFB1 is metabolized by cytochrome P450 (CYP), our aim was to develop a method to detoxify *A. flavus*-contaminated maize, under normal temperature and pressure, using *Escherichia coli* expressing human CYP3A4. First, the metabolic activity of AFB1 by recombinant human CYP3A4 was evaluated. As a result, we confirmed that recombinant human CYP3A4 metabolizes 98% of AFB1. Next, we found that aflatoxin Q1, a metabolite of AFB1 was no longer mutagenic. Furthermore, we revealed that about 50% of the AFB1 metabolic activity can be maintained for 3 months when *E. coli* expressing human CYP3A4 is freeze-dried in the presence of trehalose. Finally, we found that 80% of AFB1 in *A. flavus*-contaminated maize was metabolized by *E. coli* expressing human CYP3A4 in the presence of surfactant triton X-405 at a final concentration of 10% (v/v). From these results, we conclude that AFB1 in *A. flavus*-contaminated maize can be detoxified under normal temperature and pressure by using *E. coli* expressing human CYP3A4.

## Introduction

Aflatoxin B1 (AFB1), a kind of mycotoxin, is mainly produced by *Aspergillus flavus* (*A. flavus*) [1]. AFB1 contamination is known to occur regularly in a wide range of crops, especially in maize and nuts [2-4]. In addition, AFB1 has high thermal stability and is not decomposed under normal cooking conditions. Therefore, the effects of AFB1-contaminated food pose a significant problem to the human body [5].

To date, various abiotic control methods have been tested against aflatoxins, and some of these methods (*e.g*., using ozone, UV, clay minerals, etc.) have been reported [6, 7]. However, most of these methods have faced problems such as insufficient decomposition of AFB1, potential destruction of food components, and high cost.

Previous studies have shown that ingested AFB1 is metabolically activated by CYP3A4, which is a kind of cytochrome P450 (P450, CYP) [8]. P450 is a generic term for heme proteins that are widely present in the living world in microorganisms, animals, and plants. P450s are major drug metabolizing enzymes that bind to carbon monoxide in a reduced form and show a characteristic spectrum with an absorption maximum at 450 nm [9]. CYP3A4 is distributed in the human liver, kidney, and small intestines [10]. Furthermore, human CYP3A4 is considered to be the most important of all human P450s because it is involved in the metabolism of 45-60% of all drugs [11-14]. For example, apart from AFB1 metabolic activation, CYP3A4 is involved in the metabolism of endogenous steroids such as cortisol, testosterone, and estradiol [15].

Studies have shown that when AFB1 is metabolized by human CYP3A4, aflatoxin Q1 (AFQ1), which has relatively low toxicity, and AFB1-8,9-epoxide (AFBO), which is highly reactive with DNA and proteins, are produced [16]. AFBO is extremely dangerous because it causes cytotoxicity, mutagenicity, and carcinogenicity in vivo. On the other hand, AFBO is rapidly hydrolyzed spontaneously and is converted to AFB1-8,9-dihydrodiol, which has relatively low toxicity in vitro [16].

In this study, we aimed to metabolize and detoxify AFB1 in maize contaminated with *A. flavus* under normal temperature and pressure by using *Escherichia coli* (*E. coli*) expressing human CYP3A4. In addition, we attempted to improve the long-term storage stability of *E. coli* expressing human CYP3A4 as a biocatalyst at room temperature by evaluating the effects of disaccharides added during freeze-drying. Finally, we investigated the effect of nonionic detergents on the efficiency of AFB1 degradation by *E. coli* expressing human CYP3A4.

## Materials and Methods

AFB1 was purchased from Toronto Research Chemicals (Canada), and AFM1 was purchased from LKT Laboratories Inc. (USA). We purchased 5-methoxypsoralen (5-MOP) from Tokyo Chemical Industry Co., Ltd. (Japan).

Tryptone, dry yeast extract, Ampicillin sodium (Amp), and isopropyl-β-D-thiogalactopyranoside (IPTG) were purchased from Nacalai Tesque, Inc. (Japan). Sodium chloride (NaCl) and 5-aminolevulinic acid (5-ALA) were purchased from Kanto Chemical Co., Inc. (Japan) and Cosmo Bio Inc. (Japan), respectively.

Trehalose, sucrose, potato dextrose agar (PDA) medium, and Triton X-405 were purchased from Nacalai Tesque, Inc. (Japan). Acetonitrile, formic acid, and methanol were purchased from Wako Pure Chemical Industries, Ltd. (Japan), and syringe filters (Millex-LH) were purchased from Millipore (USA). AFLAKING was purchased from Horiba, Ltd. (Japan)*. A. flavus* (ATCC 200026, NRRL 3357) was provided by RIKEN BioResource Center (Japan). Pressure-biased maize for feed was purchased from Iisaka Seibaku Co., Ltd. (Japan). Other reagents were purchased from Nacalai Tesque, Inc. or Wako Pure Chemical Industries, Ltd.

### Culture of *E. coli* Expressing Human CYP3A4

An expression plasmid for human CYP3A4, named pCW3A4, was prepared as described previously [17] and transformed into recombinant *E. coli* (JM109 strain). The transformed *E. coli* was then inoculated into 3 ml of Luria-Bertani (LB) medium containing 50 μg/ml Amp, and cultured at 37°C, 170 rpm for 16 to 18 h. Thereafter, 1 ml of the culture solution was added to 100 ml of 2×YT medium containing 50 μg/ml Amp and cultured at 37°C and 170 rpm for 3 h. Once the optical density at 600 nm (OD_600_) of the culture reached 0.2-0.6, overexpression of the human CYP3A4 was induced by adding 100 μl of 0.5 M 5-ALA and 100 μl of 1 M IPTG. Subsequently, after culturing at 25°C and 170 rpm for 24 h, *E. coli* cells were harvested by centrifugation at 4°C and 7,000 ×*g* for 10 min. After removing the supernatant, the cell pellet was resuspended in a stock buffer (20% (v/v) glycerol, 0.1 M KPB, 1 mM EDTA) to obtain *E. coli* cells that express human CYP3A4. Expression of human CYP3A4 was calculated based on a previously developed method [18].

### AFB1 Metabolism by Human CYP3A4

*E. coli* expressing human CYP3A4 was diluted in stock buffer to an OD_600_ of 1.5. Next, we prepared the sample containing 1 ml of *E. coli* fraction diluted in stock buffer and 10 μl of 800 μM AFB1. This sample was incubated in a shaking incubator (170 rpm) at 25°C for 24 h. Thereafter, 4 ml of ethyl acetate containing 10 μM 5-MOP as an internal standard substance was added to the sample, and then this sample was vortexed and centrifuged at 25°C and 1,000 ×*g* for 10 min. Subsequently, 250 μl of the supernatant separated by centrifugation was dried under reduced pressure using a centrifugal concentrator (VC-36N/TAITEC). The sample was dissolved in 150 μl of acetonitrile, filtered with a syringe filter (Millex-LH 0.45 μm/MILLIPORE), and subjected to high performance liquid chromatography (HPLC) analysis using an HITACHI HPLC D7000 System (Japan) at excitation and detection wavelengths of 365 nm and 254 nm, respectively. We used an ODS-HPLC column (4.6 × 150 mm TSK- GEL ODS-80Ts; TOSOH, Japan) with A-Milli-Q water with 0.1% formic acid and B-acetonitrile with 0.1% formic acid for the eluent. The HPLC was run at 40°C with a flow rate of 1 ml/min with the following gradient conditions: 0 min (A: 75%) → 20 min (A: 0%) → 22 min (A: 0%) → 22.1 min (A: 75%) → 25min (A: 75%).

### LC-MS Analysis of AFB1 Metabolite

The experiment investigating the metabolism of AFB1 by the *E. coli* cells expressing human CYP3A4 was repeated three times. Each metabolite was recovered in test tubes, and then concentrated using a rotary evaporator. After purification of the AFB1 metabolite, the structure of the target metabolite was analyzed by liquid chromatography-mass spectrometry (LC-MS). LC-MS analysis was carried out using a Paradigm MS2 HPLC system (Beckman, USA) at 35°C with a 150 mm × 0.1 mm Magic C18AQ column (Michrom Bioresources, USA). The mobile phase was composed of solution C (water : acetonitrile (2 : 98) containing ammonium formate (0.1%)) and solution D (water : acetonitrile (90 : 10) containing ammonium formate (0.1%)). The gradient used was 95% solution C from 0.0 to 10.0 min; 95-45% solution C from 10.1 to 30 min; 45-10% solution C from 30.1 to 33 min; and 95% solution C from 33.1 to 45 min (equilibration). The flow rate was set to 0.5 μl/min and sample volumes of 0.5 μl were injected. MS was performed using an LTQ Orbitrap Discovery (Thermo Fisher Scientific, Inc., USA). The ESI source parameters were set as follows: ion spray voltage 1.8 kV, capillary temperature 200°C, and source heater temperature 300°C. The Orbitrap analyzer scanned the mass range from m/z 150 to 600. Data recording and processing were performed using the Xcalibur 2.1 software (Thermo Fisher Scientific, Inc.).

### Evaluation of Mutagenicity by Umu-Test

The experiment was carried out as previously described [19]. Briefly, *Salmonella* Typhimurium NM2009 was inoculated into 3 ml of TGA medium (1% (w/v) tryptone, 0.5% (w/v) NaCl, 0.2% (w/v) glucose) supplemented with Amp to a final concentration of 20 μg/ml and cultured overnight at 37°C with shaking at 140 rpm. The culture was then diluted to an OD_600_ value of 0.025 and 1 μl of the test compound was added in a 96-well microplate. Subsequently, 99 μl of 90:10:1 mixed solution of diluted bacterial solution, Cofactor1, and rat S9 fraction were added. Each sample was incubated at 37°C for 2 h. Next, 100 μl of color-developing solution was added and incubated at 37°C for 30 min, then 100 μl of 1 M sodium carbonate was added to each well and set aside for 10 min. The absorbance of each sample at 630 nm was measured using a microplate reader (MTP-810 microplate reader; CORONA, Japan).

### Freeze-Drying Treatment of *E. coli* Expressing Human CYP3A4

As described previously, *E. coli* expressing human CYP3A4 was cultured in 100 ml of 2×YT medium. *E. coli* expressing human CYP3A4 was suspended in 100 mM trehalose, 100 mM sucrose or sterile water. Thereafter, 5 ml each was dispensed, frozen with liquid nitrogen, and freeze-dried using a freeze dryer (FD-5N; Tokyo Rikakikai Co, Ltd., Japan). The sample was then stored at 25°C. After storage for each tested time period, the sample was resuspended in KPB containing 1 mM EDTA and diluted to an OD_600_ value of 1.5 and AFB1 metabolic activity was evaluated.

### AFB1 Metabolism Experiment Using *A. flavus*-Contaminated Maize

*A. flavus* was inoculated in 30 ml of potato dextrose agar (PDA) medium and incubated at 27°C for 1 week [20]. Thereafter, 20 g of pressure-biased maize for feed was added, and further cultured at 20°C for 1 week to generate *A. flavus*-contaminated maize. Subsequently, the *A. flavus*-contaminated maize was sterilized by autoclaving. Next, after weighing 17 g of contaminated maize, 200 ml of *E. coli* expressing human CYP3A4, adjusted to OD_600_ 1.5, was added to each sample and incubated at 25°C while shaking at 120 rpm for 24 h. *A. flavus* and *E. coli* expressing human CYP3A4 in each contaminated maize sample were sterilized by autoclaving. After sterilization, each contaminated maize sample was dried at 70°C for about 4 days. After crushing the contaminated maize with a coffee grinder, 34 ml of extraction solvent (acetonitrile : water = 9 : 1) and 50 μl of 100 μM AFM1 as an internal standard were added. Each contaminated maize sample was shaken at 200 rpm for 1 h at 25°C. The extract was filtered using filter paper (01511125; ADVANTEC, Japan), and the resulting solution was diluted 5-fold with sterilized water, and then filtered using glass fiber filter paper (36261090; ADVANTEC). The obtained filtrate was used as a sample diluted solution, and aflatoxins in the sample were concentrated and purified by using an immunoaffinity column (AFLAKING, HORIBA, Japan) according to the manufacturer’s instructions. The same method was used to evaluate the effect of Triton X-405 on AFB1 metabolism by *E. coli* expressing human CYP3A4. The sample was dried under reduced pressure using a centrifugal concentrator and redissolved in 150 μl of acetonitrile. Then, the sample was filtered through a syringe filter before being subjected to HPLC analysis. The analysis conditions are detailed in section “AFB1 Metabolism by Human CYP3A4.”

## Results and Discussion

### Expression of Human CYP3A4 in *E. coli*

**Fig. 1:**
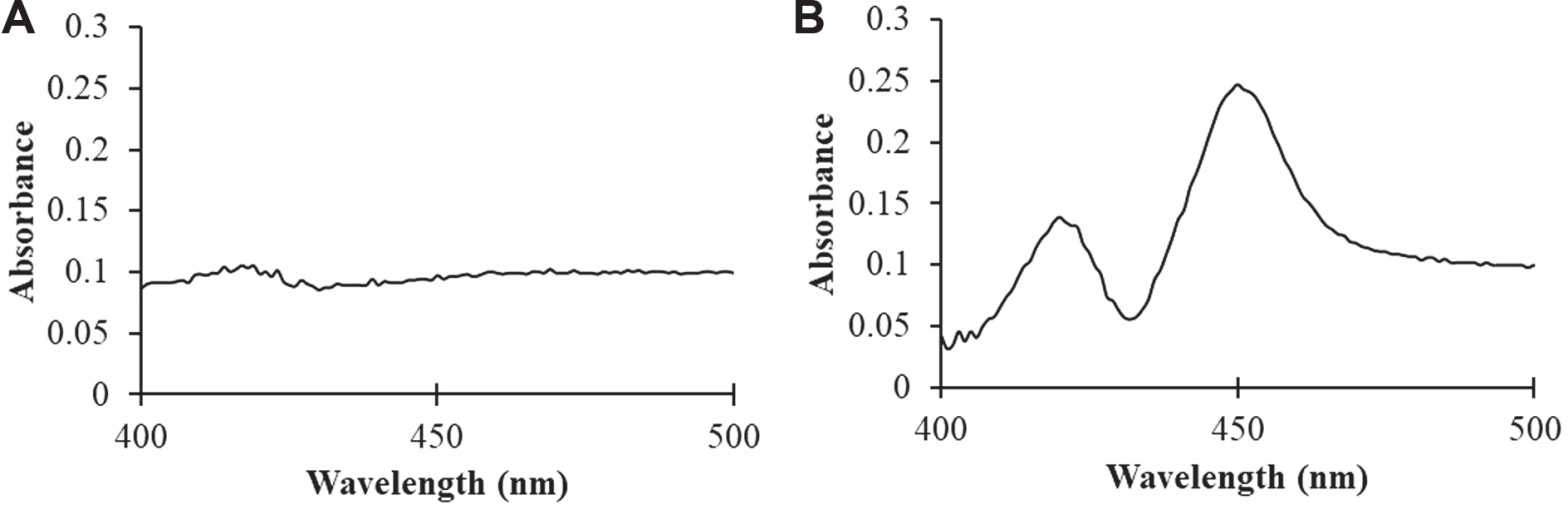
CO spectrum difference of in *E. coli* expressing human CYP3A4. Human CYP3A4 was expressed in *E. coli* and the difference in the CO spectrum was measured. The x and y axes represent the wavelength and absorbance, respectively. (**A**) *E. coli* expressing pCW (vector control, control plasmid without CYP3A4 cDNA) and (**B**) *E. coli* expressing human CYP3A4.

We transformed *E. coli* with a bacterial expression vector named pCW3A4. Thereafter, *E. coli* was cultured, and the CO difference spectrum was measured. As a result, an absorption peak at 450 nm was observed after CO treatment, suggesting that the human CYP3A4 enzyme was expressed and active in *E. coli* (Fig. 1).

### Evaluation of AFB1 Metabolism by *E. coli* Expressing Human CYP3A4

**Fig. 2:**
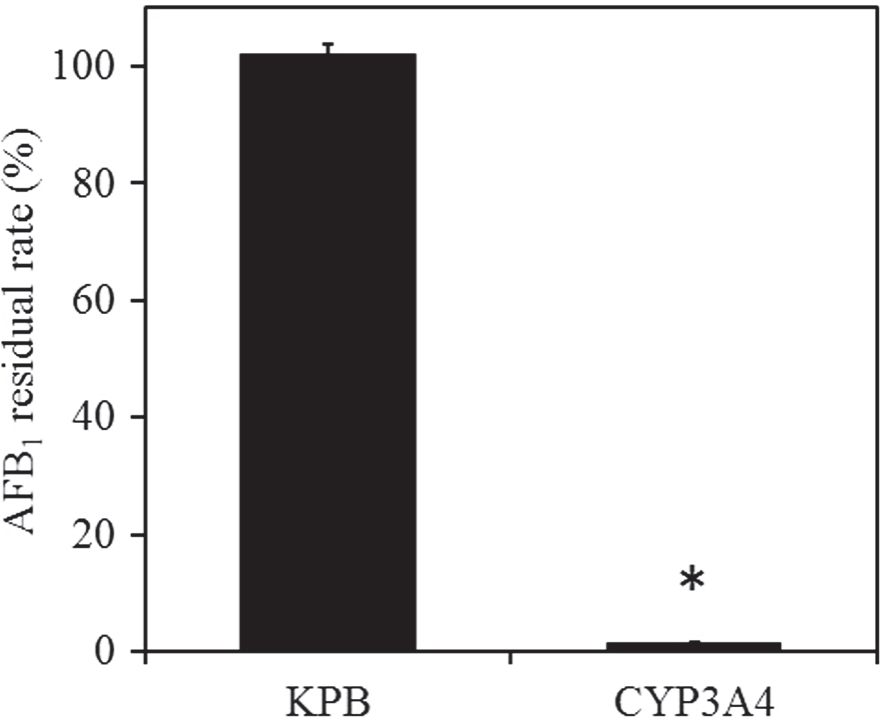
Effect of AFB1 degradation by *E. coli* expressing human CYP3A4. Metabolism experiment of AFB1 standard was performed using *E. coli* expressing human CYP3A4, and the remaining amount of AFB1 was measured. The y axis shows the relative residual rate when the amount of AFB1 (2.5 μg/ml) is 100%. *p < 0.01 versus the treatment with KPB (*n* = 3) KPB:100 mM phosphate buffer (pH 7.5) CYP3A4: 100 mM phosphate buffer (pH 7.5) and *E. coli* expressing human CYP3A4 (OD_600_ = 1.5).

Here, 1 × KPB (Control) or *E. coli* expressing human CYP3A4 was incubated with AFB1 for 24 h, and the remaining amount of AFB1 was measured by HPLC. In this way, we were able to confirm that at the start of the reaction, there was a 98% reduction in the amount of AFB1 when incubated with *E. coli* expressing human CYP3A4 compared to the control (Fig. 2).

### Identification of Human CYP3A4 AFB1 Metabolites

**Fig. 3:**
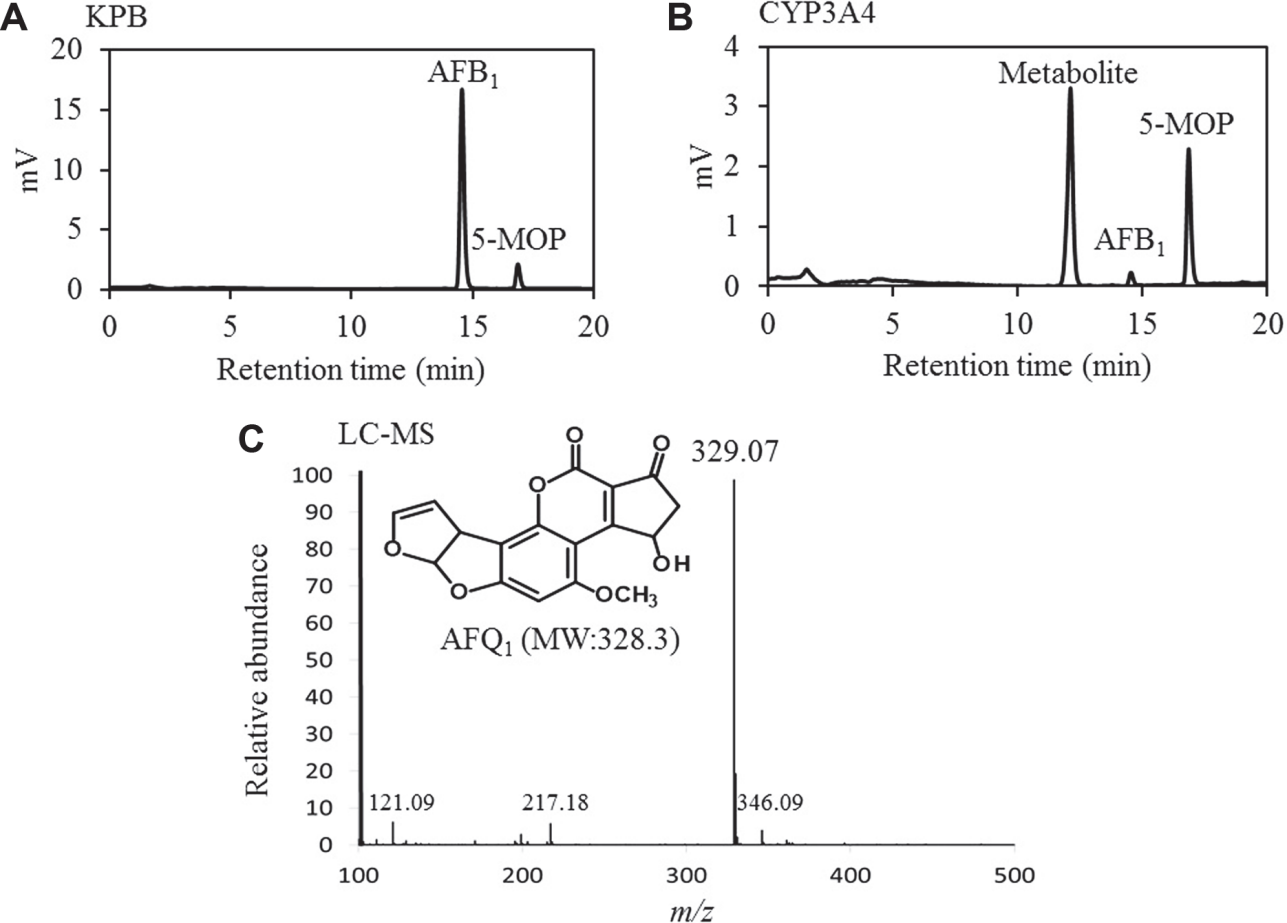
Metabolite identification by LC-MS. We conducted metabolic experiments using *E. coli* expressing human CYP3A4 and analyzed the metabolites by HPLC. (**A**) KPB, (**B**) CYP3A4. (**C**) The peak of the metabolite obtained in (B) was collected and identified by LC-MS.

In order to identify the resulting metabolites following human CYP3A4 metabolism of AFB1, the HPLC peak having a retention time of 12 to 13 min which corresponded to the metabolite was collected and analyzed by LC- MS (Fig. 3B). The [M+H]^+^ ions were detected at m/z 329 (Fig. 3C) and used as parent ions for AFQ1 [21]. We found that the main metabolite identified in this *E. coli* CYP3A4 expression system was AFQ1. AFB1 exo-8,9- epoxide easily intercalates into DNA and protein. Johnson *et al*. reported that AFB1 exo-8,9-epoxide reacts spontaneously with H2O at a pseudo-first order rate of 0.6 s^-1^ at 25°C [22]. Therefore, it is considered that AFB1 exo-8,9-epoxide was not observed as a metabolite. Furthermore, it was thought that AFBO was not detected due to the low amount of AFB1 exo-8,9-epoxide produced by CYP3A4. This is consistent with previous studies which have shown that AFB1 is mainly metabolized to AFQ1 by CYP3A4 [23].

### Evaluation of Mutagenicity of Human CYP3A4 AFB1 Metabolites

We evaluated the mutagenicity of human CYP3A4 AFB1 metabolites using the *umu*-test. We found that mutagenicity occurred with 16 μM AFB1 but not with the metabolite, AFQ1 (Table 1). Our results show that AFQ1 produced by metabolism of AFB1 by *E. coli* expressing human CYP3A4 is not mutagenic, even without undergoing additional metabolic activation. Therefore, *E. coli* expressing human CYP3A4 has the detoxifying effect of AFB1.

### Effect of Freeze-Drying and Long-term Storage of *E. coli* Expressing Human CYP3A4 on AFB1 Metabolic Activity

**Fig. 4:**
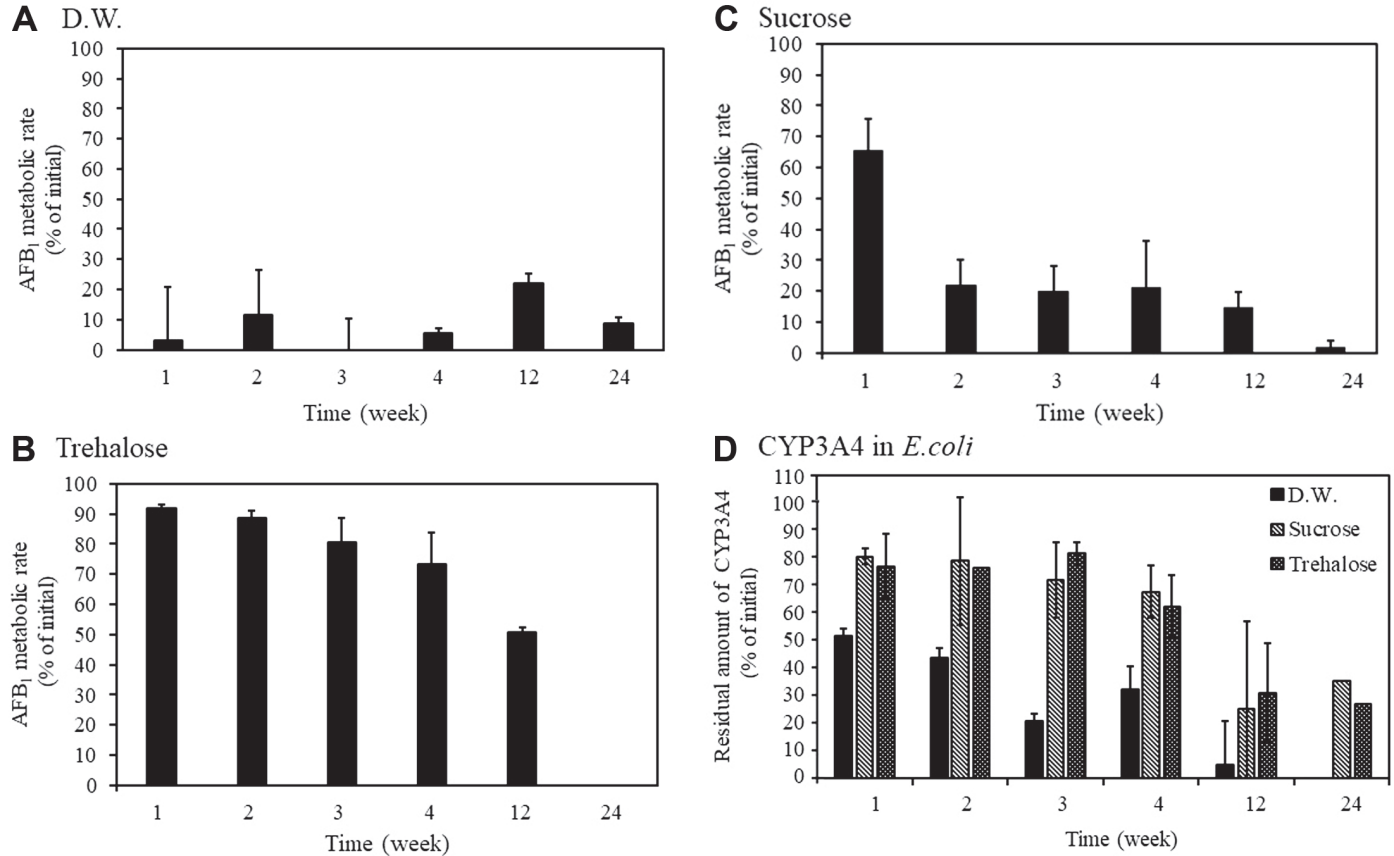
Effect of freeze-drying and long-term storage of *E. coli* expressing human CYP3A4 on AFB1 metabolic activity. After freeze-drying, metabolism experiments of the AFB1 standard and evaluation of CYP3A4 amount in *E. coli* were performed using *E. coli* expressing human CYP3A4 stored at room temperature for a certain period of time. The y axis represents the AFB1 metabolic rate when the amount of AFB1 (2.5 μg/ml) is 100%. (**A**) D.W.: freeze-dried with sterilized water, (**B**) Sucrose: freeze-dried with sucrose, (**C**) Trehalose: freeze-dried with trehalose. (**D**) CYP3A4 in *E. coli*: The y axis represents the residual amount of CYP3A4 in *E. coli* when the starting amount of CYP3A4 (3.2 pmol in *E. coli* suspension) is 100%.

We expect that the usefulness of this AFB1 detoxification system will be enhanced if *E. coli* expressing human CYP3A4 can be stored for a long period of time and still maintain its activity at room temperature. Therefore, we selected freeze-drying as the preservation method for *E. coli* expressing human CYP3A4, and investigated the effect of different protective agents and storage periods. We found that when a protective agent was not used, AFB1 metabolic activity decreased by more than 95% in 7 days (Fig. 4A). Therefore, *E. coli* expressing human CYP3A4 cannot be stored for a long period of time under normal conditions. In addition, when sucrose was used, AFB1 metabolic activity could not be maintained for a long time (Fig. 4B). On the other hand, when trehalose was used as a preservative, the AFB1 metabolic activity after 3 months was about 50% of that at the start of preservation (Fig. 4C).

In general, freeze-drying treatment may cause changes in cell membrane structure or protein structure in *E. coli*. Disaccharides such as trehalose and sucrose, are known to prevent the destruction of the lipid membrane structures by substituting the water between the lipid heads of the cell membrane during freeze-drying [24], thereby preventing degeneration by forming hydrogen bonds with the protein [25]. When we measured amount of CYP3A4 after freeze-drying treatment, about 50% of the CYP3A4 was degraded in one week without protective materials. On the other hand, when trehalose and glucose were used as protective materials, more than 60% of P450 remained undegraded after 4 weeks (Fig. 4D). Therefore, we propose that the addition of trehalose stabilized the membrane structure and CYP3A4 expressed in *E. coli*, allowing the preservation of freeze-dried AFB1 metabolic activity over time. On the other hand, it was also shown that glucose has an effect of protecting CYP3A4 expressed in *E. coli* from degradation (Fig. 4D). A previous study with *Bacillus thuringiensis*, which had been freeze-dried with 100 mM trehalose, showed improved resistance to humidity and air exposure. Although improvements in resistance to these environmental factors cannot be confirmed when sucrose is used in the freeze-drying process [26], these results suggest that selection of the optimal protective agent is important for AFB1 metabolism by recombinant human CYP3A4.

### AFB1 Metabolism in *A. flavus*-Contaminated Maize Using *E. coli* Expressing Human CYP3A4

**Fig. 5:**
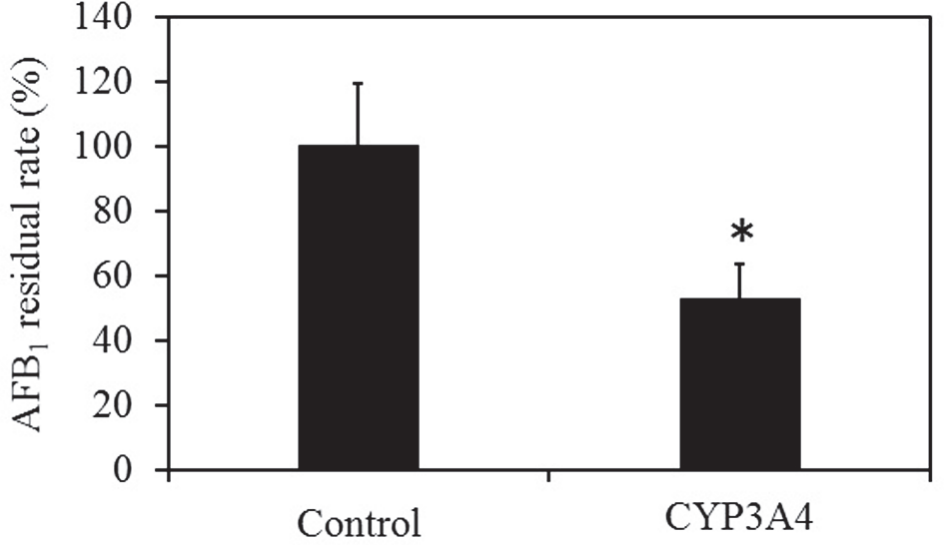
Metabolic experiment using *A. flavus*-contaminated maize. Metabolic experiments of AFB1 in *A. flavus*- contaminated maize were conducted using *E. coli* expressing human CYP3A4, and the residual amount of AFB1 in each sample was measured. The graph shows the relative residual rate when the residual amount of untreated (Control) is 100%. *p < 0.05 versus control (*n* = 3). Control: untreated CYP3A4: 100 mM phosphate buffer (pH 7.5) and *E. coli* expressing human CYP3A4 (OD_600_ = 1.5).

Our aim here was to investigate whether AFB1 in *A. flavus*-contaminated maize can be metabolized by recombinant human CYP3A4. We found that when *A. flavus*-contaminated maize was treated with *E. coli* expressing human CYP3A4, there was a ~50% decrease in AFB1 compared to the control (Fig. 5). Moreover, when a metabolic experiment was performed using the AFB1 standard, there was a concomitant reduction in AFB1 and mutagenicity (Fig. 2, Table 1). Therefore, recombinant human CYP3A4 detoxifies AFB1 in *A. flavus*-contaminated maize under normal temperature and pressure without affecting nutritional value.

**Fig. 6:**
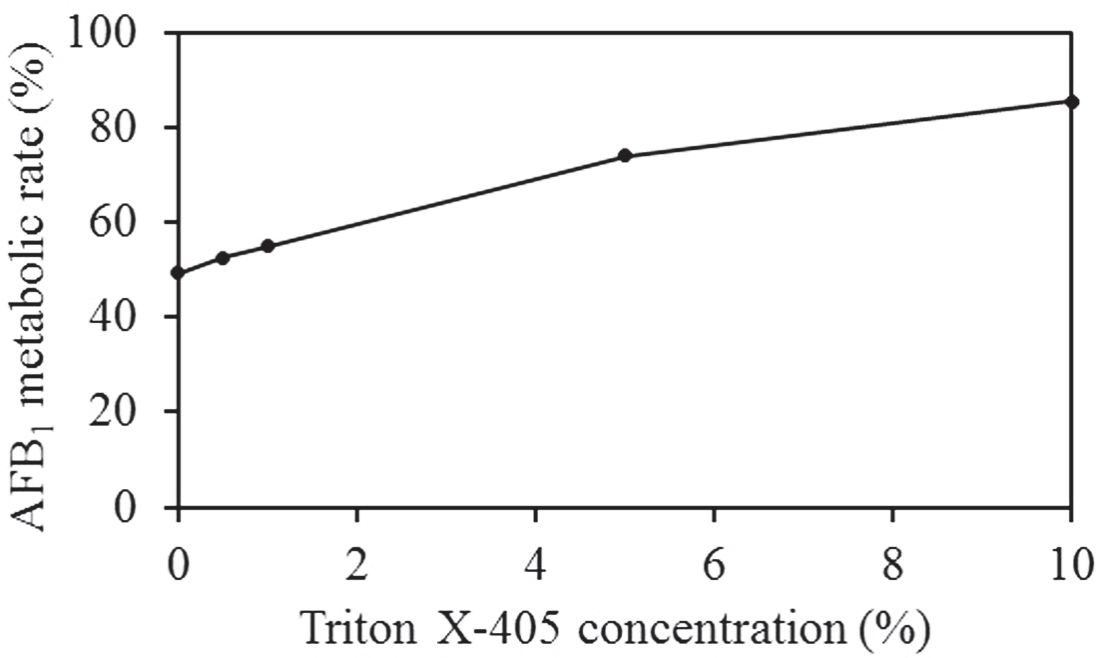
Effect of Triton X-405 on AFB1 metabolism by *E. coli* expressing human CYP3A4. By changing the concentration of Triton X-405, AFB1 in *A. flavus*-contaminated maize was metabolized using *E. coli* expressing human CYP3A4, and the metabolic rate of AFB1 was measured. This graph shows the change in the AFB1 metabolic rate when the residual amount of untreated (Control) is 100%.

A previous study by [27] reported that the control method using lactic acid bacteria reduces the total amount of aflatoxins in contaminated foods by binding aflatoxins to the cell walls. Therefore, in this study, we also wanted to investigate the possibility of metabolizing more AFB1 by adjusting the binding of AFB1 to *E. coli* expressing human CYP3A4. In order to further improve the efficiency of detoxification by *E. coli* expressing human CYP3A4, a metabolic experiment was conducted using the surfactant, Triton X-405. We found that AFB1 metabolism increased with the addition of Triton X-405 in a concentration-dependent manner. Additionally, 80% of the AFB1 in contaminated maize disappeared when 10% (w/v) Triton X-405 was added (Fig. 6). Since it is a surfactant, we hypothesize that Triton X-405 relaxes the cell membranes of *A. flavus* and *E. coli* resulting in the formation of micelle structures and improved water solubility of chemical substances. Previously, Schnaitman has shown that Triton X-100, which has a similar structure to Triton X-405, relaxes the membrane structure of *E. coli* but does not affect the normal morphology of the cell wall [28]. Therefore, we presume that the addition of Triton X-405 relaxes the cell membranes of *E. coli* cells, thereby increasing the incorporation of solubilized AFB1 into the cell and resulting in improved AFB1 metabolic efficiency by human CYP3A4. Recently, Chen *et al.* also reported that the addition of nonionic surfactants alters the structure of cell membranes of *Monascus* fungi and promotes the secretion of *Monascus* pigments, a natural food coloring [29].

In this study, we optimized the conditions for reacting *A. flavus*-contaminated maize and *E. coli* expressing human CYP3A4 in vitro under normal temperature and pressure. As a result, we succeeded in removing about 80% of AFB1 from *A. flavus*-contaminated maize. Therefore, it became clear that this method can be used for metabolism and detoxification of AFB1 in food. This is the first report of AFB1 detoxification from maize contaminated with *A. flavus*. In the future, it will be important to investigate the nutritional components of maize after CYP3A4 metabolism.

## Figures and Tables

**Table 1:** **Evaluation of AFB_1_ and AFQ_1_ mutagenicity by *umu*-test.** *Umu*-test was performed using AFB_1_ or AFQ_1_, and the rat S9 fraction. The relative β-galactosidase activity is shown when the absorbance at AFB_1_ 0 μM (DMSO) is 1, and those with more than twice the activity were evaluated as mutagenic.

Chemical	S9 (+/-)	Concentration (μM)	β-Galactosidase activity (Relative absorbance)	Mutagenicity (+/-)
AFB1	-	0	1.00 (0.09)	-
AFB1	+	0	1.00 (0.04)	-
AFB1	-	16	1.23 (0.12)	-
AFQ1	+	16	0.70 (0.07)	-
AFB1	-	16	2.25 (0.16)	+
AFQ1	+	16	1.30 (0.10)	-
